# Cellular Capacities for High-Light Acclimation and Changing Lipid Profiles across Life Cycle Stages of the Green Alga *Haematococcus pluvialis*

**DOI:** 10.1371/journal.pone.0106679

**Published:** 2014-09-15

**Authors:** Baobei Wang, Zhen Zhang, Qiang Hu, Milton Sommerfeld, Yinghua Lu, Danxiang Han

**Affiliations:** 1 Department of Chemical and Biochemical Engineering, Xiamen University, Xiamen, Fujian, China; 2 Department of Human System and Environment, Arizona State University, Mesa, Arizona, United States of America; 3 State Key Laboratory of Bioreactor Engineering, East China University of Science and Technology, Shanghai, China; 4 Center for Microalgal Biotechnology and Biofuels, Institute of Hydrobiology, Chinese Academy of Sciences, Wuhan, Hubei, China; Mount Allison University, Canada

## Abstract

The unicellular microalga *Haematococcus pluvialis* has emerged as a promising biomass feedstock for the ketocarotenoid astaxanthin and neutral lipid triacylglycerol. Motile flagellates, resting palmella cells, and cysts are the major life cycle stages of *H. pluvialis*. Fast-growing motile cells are usually used to induce astaxanthin and triacylglycerol biosynthesis under stress conditions (high light or nutrient starvation); however, productivity of biomass and bioproducts are compromised due to the susceptibility of motile cells to stress. This study revealed that the Photosystem II (PSII) reaction center D1 protein, the manganese-stabilizing protein PsbO, and several major membrane glycerolipids (particularly for chloroplast membrane lipids monogalactosyldiacylglycerol and phosphatidylglycerol), decreased dramatically in motile cells under high light (HL). In contrast, palmella cells, which are transformed from motile cells after an extended period of time under favorable growth conditions, have developed multiple protective mechanisms—including reduction in chloroplast membrane lipids content, downplay of linear photosynthetic electron transport, and activating nonphotochemical quenching mechanisms—while accumulating triacylglycerol. Consequently, the membrane lipids and PSII proteins (D1 and PsbO) remained relatively stable in palmella cells subjected to HL. Introducing palmella instead of motile cells to stress conditions may greatly increase astaxanthin and lipid production in *H. pluvialis* culture.

## Introduction

Astaxanthin is a superb antioxidant and a natural food coloring agent that has been used in nutraceutical, aquaculture, and poultry industries [1], [2]. Among the naturally occurring organisms capable of producing astaxanthin, the unicellular microalga *Haematococcus pluvialis* can accumulate the largest amounts [up to 4% of its dry weight (DW)] under various adverse environmental or culture conditions [3]. Over the past two decades, mass culture of *H. pluvialis* in photobioreactors has been exploited to produce natural astaxanthin [4], [5]. Recently, this organism has also emerged as a promising cell factory for biofuels because of its ability to produce large amounts of neutral lipids, mainly in the form of triacylglycerol (TAG) [6]. TAG is a feedstock for the production of biofuels like biodiesel and bio–jet fuel, bioplastics, and other chemicals that are currently derived from fossil fuels. Moreover, because of its great growth potential and high photosynthetic efficiency, *H. pluvialis* is an alternative solution for removing CO_2_ from fossil-fired power plants [7].

A two-stage cultivation strategy is often applied to mass culture of *H. pluvialis* [8]–[10]. In the green stage, optimal light intensity and nutrient-replete media are provided to promote the growth of green vegetative cells; when the cell density reaches a maximal level, the culture is subjected to stress conditions to induce astaxanthin biosynthesis and accumulation. At this red stage, many cells die off, while the surviving ones undergo profound biochemical and cellular changes, transforming the flagellates (i.e., vegetative cells) into red cysts (aplanospores). Although cell death is related to high light (HL), high salinity, and other stressors, such as the application of acetate or Fe^2+^ to the cultures [9], [11]–[14], the exact causes of cell death under stress remained largely unknown. The susceptibility of fast-growing *H. pluvialis* cells to adverse culture conditions leads to a substantial reduction in biomass productivity, a major obstacle that prevents expansion of the *H. pluvialis* industry.

It has recently been observed that the *H. pluvialis* strain CCAP 34/12, which is dominated by flagellates at the exponential growth phase, was more susceptible to HL stress than another strain (SAG 34/1b) dominated by resting vegetative cells. These resting cells are also called palmella cells and are transformed from flagellates under favorable growing conditions. The death of flagellates under HL was attributed to the production of reactive oxygen species (ROS) [11]. Although a number of protective mechanisms contributing to the survival of SAG 34/1b under HL were identified, including down-regulation of linear photosynthetic electron transport and enhancement of the alternative plastid terminal oxidase pathway, it was unclear whether these mechanisms were developed during the cell transformation or resulted from different genetic makeups of the two *Haematococcus* strains. A recent comparative proteomic analysis of flagellates and resting (palmella) cells from a single *Haematococcus* strain showed that a number of proteins involved in stress responses were induced in the resting cells but absent in the flagellates [15].

The aim of this study was to determine the physiological and biochemical changes that occur during the transformation of motile flagellates into resting palmella cells and to dissect the key mechanisms by which the different forms of *Haematococcus* cells cope with HL. To gain more insight into the molecular–level changes in lipids that occur in response to HL, we developed a mass spectrometry–based lipidomics method for absolute quantification of glycerolipids. Our results suggest that introducing resting palmella instead of motile flagellates into mass culture represents a promising strategy to increase the production of biomass and bioproducts from *H. pluvialis*.

## Materials and Methods

### Culture and strains

*Haematococcus pluvialis* NIES144 was obtained from the National Institute for Environmental Studies in Tsukuba, Japan. Algal cells were grown in 2.8-L flasks containing 1 L basal growth medium [16] at 22°C under continuous low light (LL) illumination (20 µmol photons m^−2^ s^−1^). Cultures were maintained in a resting culture mode with manual shaking once per day. To obtain motile cells, cultures were maintained under the above culture conditions for 4 days, at which point ca. 70% of cells were motile cells and the remainder were palmella cells; the flagellates suspended in the growth medium were then harvested and enriched with 98.0±1.8% motile cells. Palmella cells were predominant in the cultures maintained under the above conditions for 7 days. To increase the quantity of palmella cells, cells that settled at the bottom of the flasks were collected and washed with fresh growth medium five times to remove any remaining motile cells. Motile and palmella cells were both exposed to continuous high light (HL) illumination (150 µmol photons m^−2^ s^−1^) for 24 h. Red cyst cells transformed from motile cells are referred to as RC-M, and those induced from palmella cells are termed RC-P. Algal cells were harvested by centrifugation at 1,000 g for 10 min. Aliquots of fresh cell pellets were resuspended in the breaking buffer [5.0 mM HEPES, 0.3 M sorbitol, and 1% protease inhibitor cocktail (Sigma-Aldrich, USA)] for immunoblotting, or growth medium for chlorophyll fluorometry analysis. Freeze-dried algal biomass were used for biochemical composition (e.g. pigments, lipids, proteins and carbohydrates) analyses. Each type of cells was prepared in triplicate.

### Carotenoids and chlorophyll analysis

Canthaxanthin, astaxanthin, *β*-carotene, chlorophyll *a* (chl*a*), and chlorophyll *b* (chl*b*) contents were analyzed by high-performance liquid chromatography (HPLC) according to the method described previously [17].

### Photosynthetic measurements

Pulse amplitude modulated chl*a* fluorimetric analysis was conducted using the Dual-PAM-100 system (Heinz Walz, Germany). Harvested cells were resuspended in fresh growth medium and were dark-adapted for 15 min before measurement. One mL samples were loaded into 1 cm cuvettes and stirred gently with a magnetic stir bar. The minimal fluorescence (F_0_) was recorded under the measuring light, and after ∼5–10 s, a saturated pulse light (∼10,000 µmol photons m^−2^s^−1^ lasting for 0.8 s) was applied to fully close the PSII reaction centers to measure the maximum fluorescence (F_m_) [18]. A series of actinic light (30∼849 µmol m^−2^ s^−1^) were switched on, and at intervals of 35 s, saturating flashes were applied. From this Saturation Pulse analysis, the maximum fluorescence in the light (Fm′) and steady-state value of fluorescence (F) were recorded. The following parameters indicating photosynthetic efficiency and energy quenching at PSII were calculated: Fv/Fm (potential maximum quantum efficiency)  =  (F_m_−F_0_)/F_m_; Y(II) (quantum yield of PSII)  =  (F_m_′−F)/F_m_′; ETR(II)  =  Photosynthetically active radiation×0.5×0.84×Y(II); NPQ (nonphotochemical quenching)  =  (F_m_−F_m_′)/F_m_′; and Y(NO) (non-regulated energy dissipation)  =  F/Fm [19]. The photosynthesis-irradiance parameters (Ik, ETRmax, and α) were retrieved from the ETR(II)-irradiance curve by using the Eilers and Peeters model [20].

### Immunoblot analysis

Algal cells were disrupted using a mini bead-beater (Biospec, USA). The homogenates were centrifuged at 1,000 g for 3 min at 4°C to remove unbroken cells and cell debris. The supernatant was then transferred to a new tube and centrifuged at 12,000 g for 30 min at 4°C to obtain the crude membranes. The resulting pellets were resuspended in 60 µL SBA buffer containing 0.1 M dithiothreitol, 0.1 M Na_2_CO_3_, 40 µL 30% sucrose, and 5% SDS and were then vortexed at 3,000 rpm for 30 min at room temperature to extract total proteins. Insoluble proteins were removed by centrifugation at 12,000 g for 10 min at 4°C. The concentration of total membrane proteins in the supernatant was measured with a CB-X protein assay kit (G-Biosciences, USA). Proteins were separated by SDS-PAGE (4–20% precast polyacrylamide gel, Bio-Rad, USA) and transferred to nitrocellulose membranes. Primary antibodies of the D1 protein of PSII, Rieske iron-sulfur protein (RISP) of cytochrome *b*_6_ *f* complex, PsaA protein of PSI, and PsbO protein of the oxygen-evolving complex (OEC) were obtained from Agrisera (Sweden). Antigen-antibody complexes were visualized using an enhanced chemiluminescence substrate detection kit (Thermo Fisher Scientific, USA). Intensities of visualized protein bands were measured by using the program ImageJ (http://imagej.nih.gov/ij/).

### Total protein analysis

Ten mg algal biomass was incubated with 100 µL 1 M NaOH at 80°C for 10 min and then diluted with 900 µL water. Insoluble cell debris was removed by centrifugation at 12,000 g for 30 min at 4°C and was then extracted twice. The resulting supernatants from three extractions were combined for protein assays using a Bio-Rad Protein Assay (Bio-Rad, USA). Bovine serum albumin was used for calibration.

### Total carbohydrate analysis

Ten mg algal biomass was pretreated with 0.5 mL acetic acid for 20 min at 60°C to break the cell walls. Samples were immediately placed on ice, and 10 mL acetone was added to each sample to extract the pigments. The extracts were centrifuged at 1,000 g for 2 min at 4°C. The resulting pellets were resuspended in 4 mL trifluoroacetic acid and incubated in boiling water for 4 h to further disrupt the cells and extract the carbohydrates. Samples were cooled on ice before 5 mL water was added. Cell debris was removed by centrifugation at 10,000 g for 2 min at 4°C, and the supernatant was recovered for total carbohydrate measurement using the phenol-sulfuric acid method [21]. Glucose was used as standard for calibration.

### Lipid analysis and quantification with Liquid Chromatography-Mass/Mass Spectrometry

For lipidomic analysis, 10 mg lyophilized algal cells were homogenized in liquid nitrogen, and lipids were extracted with chloroform∶methanol (2∶1, *v*/*v*) [22]. Lipidomic analyses were performed on a 6460 triple quadrupole electrospray ionization mass spectrometer equipped with 1260 high performance liquid chromatography (Agilent, USA). The instrumental parameters were set up as follows: nebulizing gas (nitrogen), 40 psi; dry gas (nitrogen), 4 L min^-1^ at 200°C; spray capillary voltage, 4,000 V for the positive ion mode and 3,500 V for the negative ion mode; gas temperature, 250°C; gas flow, 5 mL min^−1^; and sheath gas temperature, 350°C. Chloroplast membrane lipids, including monogalactosyldiacylglycerol (MGDG), digalactosyldiacylglycerol (DGDG), sulfoquinovosyldiacylglycerol (SQDG) and phosphatidylglycerol (PG), were identified by precursor ion scanning for lipid ions, which yielded the diagnostic ions associated with their head groups induced by collision [23]. Phospholipids, including phosphatidylinositol (PI), phosphatidycholine (PC), phosphatidylethanolamine (PE), were identified according to the previously described, collision-induced dissociation principles developed for these lipids [24]. To identify betaine lipid diacylglycerol-O-(N, N, N-trimethyl)-homoserine (DGTS), the ion [C_10_H_22_NO_5_]^+^ (*m*/*z* 236) was used for precursor ion scanning. TAGs were identified using sequential neutral loss scanning [25]. Product ion scanning was employed to determine fatty acyl groups.

For quantitative analysis, the protonated forms ([M+H]^+^) of DGTS, PC, and PE were detected by multiple reaction monitoring (MRM) in a positive ion mode, while the ammonium adducts ([M+NH_4_]^+^) of MGDG, DGDG, and TAG were analyzed by single-stage mass spectrometry (MS). The deprotonated forms of the anionic glycerolipids PG, PI, and SQDG were analyzed by MRM in a negative mode. Prior to MS analysis, lipid extracts were separated on a ZOBAX SB C18 column (1.8 µm, i.d. 2.1 mm, l.150 mm, Agilent, USA) for the positive mode or on an Extend C18 column (1.8 µm, i.d. 2.1 mm, l.150 mm, Agilent, USA) for the negative mode. The mobile phase for the positive mode was comprised of A: methanol∶acetonitrile∶H_2_O (19∶19∶2, *v*/*v*/*v*) and B: isopropanol; both contained 10 mM ammonium acetate and 0.1% (*w*/*v*) formic acid. The following gradient was used: 0–5 min, 90% A, 10% B; 25 min, 60% A, 40% B; 60 min, 45% A, 55% B; and 62 min, 45% A, 55% B. The mobile phase for the negative mode was comprised of A: methanol: acetonitrile: water (25∶25∶8, *v*/*v*/*v*) and B: isopropanol. Both A and B contained 0.025% (*w*/*v*) ammonium hydroxide. Samples were eluted with a gradient elution solution as follows: 0 min, 100% A; 10 min, 95% A, 5% B; 12 min 45% A, 55% B; and 30 min 45% A, 55% B. For each sample, the column was re-equilibrated with A for 10 min before gradient elution. The temperature of the columns was maintained at 40°C, and the flow rate was 0.2 mL min^−1^.

For absolute quantification, lipid extracts were mixed with the internal standards (ITSD), including TAG 17∶0/17∶0/17∶0 (Sigma-Aldrich, USA), MGDG 18∶0/18∶0 (Avanti Polar Lipid, USA), DGDG 18∶0/18∶0 (Avanti Polar Lipid), PE 14∶1/17∶0 (Avanti Polar Lipid), PG 17∶0/20∶4 (Avanti Polar Lipid), PC 17∶0/20∶4 (Avanti Polar Lipid), and PI 17∶0/20∶4 (Avanti Polar Lipid). Among these, PC 17∶0/20∶4 was used as an ITSD for both PC and DGTS quantification, and PI 17∶0/20∶4 was used for both PI and SQDG quantification. The external standards (ETSD) for calibration included TAG 16∶1/16∶1/16∶1 (for TAG species containing 48 carbon atoms in three acyl chains, TAG C48), TAG 16∶0/18∶1/16∶0 (for TAG C50), TAG 18∶1/16∶0/18∶1 (for TAG C52), and TAG 18∶1/18∶1/18∶1 (for TAG C54) (Sigma-Aldrich). MGDG 16∶3/18∶3 (Matreya, USA), DGDG 18∶3/18∶3 (Matreya), PE 20∶4/20∶4 (Avanti Polar Lipid), PG 18∶0/18∶1 (Avanti Polar Lipid), PC 18∶1/18∶1 (Avanti Polar Lipid), DGTS 16∶0/16∶0 (Avanti Polar Lipid), and SQDG (Indofine Chemical, UK) were used as ETSDs for the corresponding classes of membrane lipids. ETSD were titrated relative to a constant amount of ITSD to establish the correlation between the ratio of the analyte signal to the ITSD signal and the ratio between their concentrations.

### Statistical analysis

Student's t-test was used to compare the cellular content of pigments, lipids, proteins and carbohydrates (n = 6) between given two cell forms, as well as for the semi-quantitative results of immunoblotting (n = 2). If the test gives *p* value ≤ 0.05, the differences between two cell forms were interpreted as being significant.

## Results

### Morphological changes of *Haematococcus* cells during the transformation from motile flagellates to resting palmella under low-light and high-light acclimation

**Figure 1:**
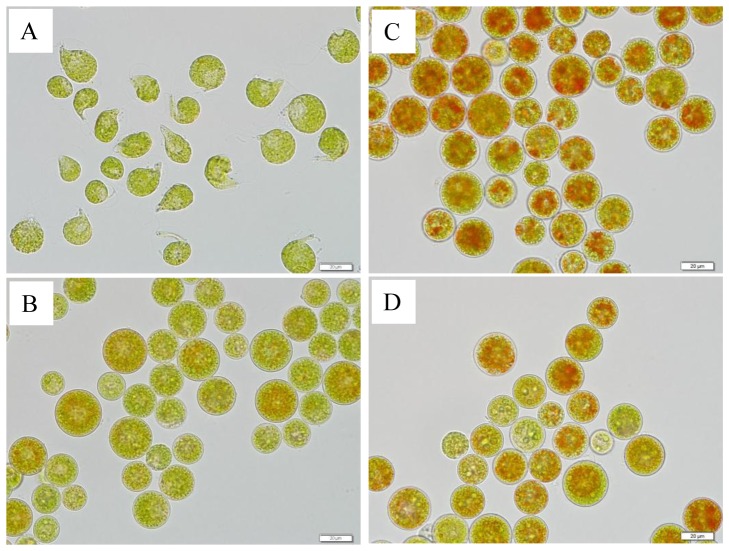
Different *H. pluvialis*cell types. (A) Motile cells grown under low light; (B) palmella cells grown under low light; (C) red cells induced from motile cells grown under high light for 24 h; (D) red cells induced from palmella cells grown under high light for 24 h.

Under the favorable LL condition, the motile cells were usually pear-shaped with a pair of flagellae at the anterior end, and the protoplast of the motile cell was enclosed by a swollen, gelatinous extracellular matrix (**Fig. 1A**). The palmella cells were spherical with rigid cell walls and were somewhat reddish in the center, indicative of the presence of astaxanthin (**Fig. 1B**). After exposure to HL for 24 h, the motile cells formed rigid, thick cell walls, and accumulated astaxanthin (**Fig. 1C**). Greater amounts of astaxanthin were evident in the palmella cells under HL than LL (**Fig. 1D**).

### Pigment profiling

HPLC analysis showed that under LL, the motile cells contained more chl *a* and chl *b* than palmella cells by 17.8% (*p*<0.01) and 22.8% (*p*<0.01), respectively. However, the ratio of chl *a* to chl *b* in these two cell types was similar. The *β*-carotene content in both cells was also similar (ca. 2.30 mg g^−1^ DW). Neither canthaxanthin nor astaxanthin was detected in motile cells, whereas the palmella cells accumulated small but noticeable amounts of these two carotenoids under LL (**Table 1**).

**Table 1:** Effect of high light intensity on pigment contents in *H. pluvialis*in different cells types.

Cell type	Chl*a*	Chl*b*	Chl*a*+*b*	*β*-carotene	Astaxanthin	Canthaxanthin	Chl*a*:*b*	β-carotene:chl
MC	24.02±0.71	4.96±0.13	28.98±0.79	2.30±0.32	ND	ND	4.85	0.08
RC-M	24.22±1.03	2.95±0.09	27.32±1.15	1.06±0.10	4.96±0.20	0.20±0.01	8.18	0.04
PC	20.39±0.89	4.04±0.19	24.38±1.04	2.25±0.25	0.66±0.06	0.24±0.01	5.11	0.09
RC-P	21.89±0.80	2.60±0.11	24.49±0.91	1.17±0.09	3.01±0.16	0.21±0.01	8.36	0.05

Pigment contents (mg g^−1^DW) were measured by HPLC. Values represent means ±SD, n = 6.

ND: not detected. MC: motile cells; PC: palmella cells; RC-M: red cells induced from motile cells; RC-P: red cells induced from palmella cells.

After exposure to HL for 24 h, chl *b* declined considerably while chl *a* remained stable in both motile and palmella cells; the ratio of chl *a* to chl *b* increased from 4.85 to 8.18 in red cysts transformed from motile cells (RC-M), and from 5.11 to 8.36 in red cysts transformed from palmella cells (RC-P) (**Table 1**). During the same period, the *β*-carotene content in motile and palmella cells decreased by 53.9% (*p*<0.01) and 48.2% (*p*<0.01), respectively. After 24 h exposure to HL, astaxanthin in RC-M equaled 4.96 mg g^−1^ DW, which was 64.7% greater than in RC-P (*p*<0.01).

### Changes in photosynthetic capacity during the transformation from motile flagellates to resting palmella under low-light and high-light acclimation

**Figure 2:**
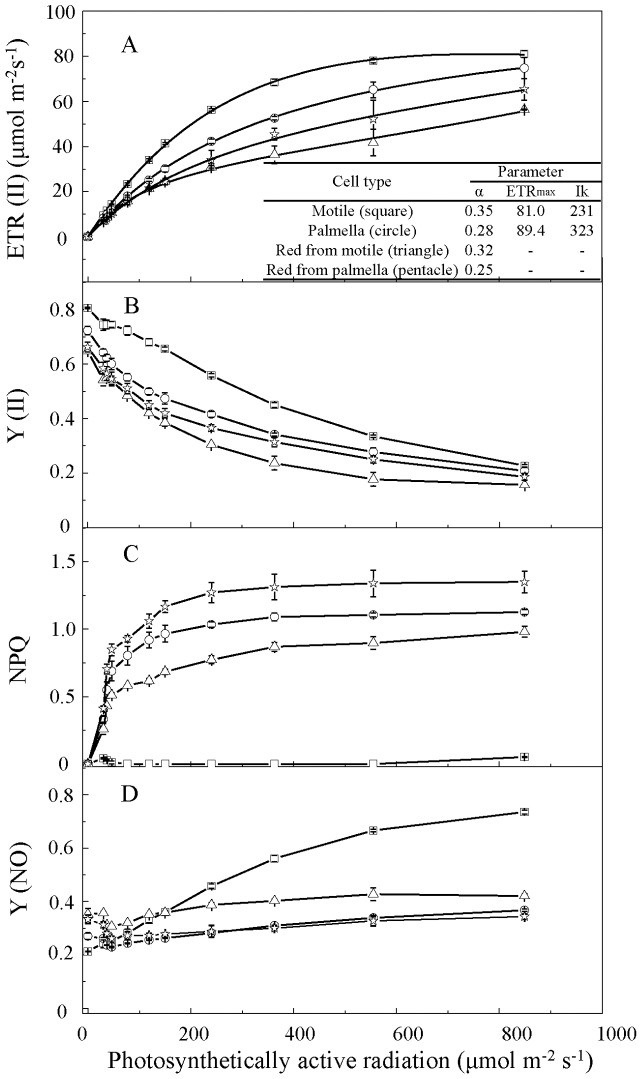
Light intensity response curves in different types of cells. (A) Photosynthetic electron transport rate in photosystem II [ETR(II)]; (B) quantum yield in photosystem II [Y(II)]; (C) nonphotochemical quenching (NPQ); (D) energy dissipated by a nonregulated mechanism in photosystem II [Y(NO)]. Values represent the mean ± S.D. (n = 3). Motile cells: square; palmella cells: circle; red cells induced from motile cells: triangle; red cells induced from palmella cells: pentacle.

To investigate the changes in photosynthetic capacity during encystment and HL acclimation, the photosynthetic efficiency of PS II of different cell forms were measured by using chlorophyll fluorometry under varying light intensities (**Fig. 2**). As shown in the ETR-irradiance curve (**Fig. 2A**), motile and palmella cells exhibited different responses to the changing light intensities. Motile cells possess an initial rate (α) higher than that of palmella cells, whereas its ETR_max_ was lower than that of palmella cells. The saturation irradiance for palmella cells is 323 µmol m^−2^ s^−1^, significantly higher than that for motile cells (231 µmol m^−2^ s^−1^). These results indicated that palmella cells may possess more pronounced capacities for high light adaptation, but motile cells can dominate in the environment with lower irradiance. The initial slope of RC-M and RC-P was slightly declined as compared to motile cells and palmella cells, respectively. For both RC-M and RC-P, ETR didn't reach a saturation level under the highest light intensity (849 µmol photons m^−2^ s^−1^) tested.

Motile cells exhibited higher Y(II) than palmella cells under light intensities of 30–555 µmol photons m^−2^s^−1^ (**Fig. 2B**), suggesting that motile cells possessed a greater ability than palmella cells to convert excited energy at PSII to photochemical energy under low and moderate light intensities. However, such a capacity was severely impaired in RC-M, of which Y(II) is significantly lower than that of RC-P, especially under the moderate light intensities (200–555 µmol photons m^−2^ s^−1^, *p*<0.05). Under the strongest irradiance (849 µmol photons m^−2^ s^−1^), no significant difference with respect to Y(II) was observed for all the cell forms.

In addition to yielding photochemical energy, a portion of the excitation energy at PSII is dissipated by a regulated, nonphotochemical quenching mechanism and a nonregulated energy dissipation [26], [27], as indicated by NPQ and Y(NO), respectively (**Fig. 2C, D**). NPQ involves the de-excitation of ^1^Chl to the ground state by the emission of excess energy as heat. This process is affected by acidification of the thylakoid lumen and the xanthophyll cycle, and is thus considered a regulatory mechanism protecting algae and higher plants from excess light [26]. Y(NO) is the yield of non-regulated energy dissipation of PSII [19]. Our results showed NPQ was absent in motile cells, whereas it was rapidly induced in palmella cells with the increase of light intensity and gradually saturated at the level of 1.0 above the irradiance of 200 µmol photons m^−2^ s^−1^ (**Fig. 2C**). Although the NPQ was augmented in both RC-M and RC-P as compared to motile cells and palmella cells, respectively, RC-P exhibited the greatest NPQ level among the four cell forms. Consequently, nonregulated energy dissipation [Y(NO)] was maintained at a relatively low level in palmella cells and RC-P under all the irradiances, whereas in motile cells it increased remarkably in response to the increased light intensities (**Fig. 2D**).

**Figure 3:**
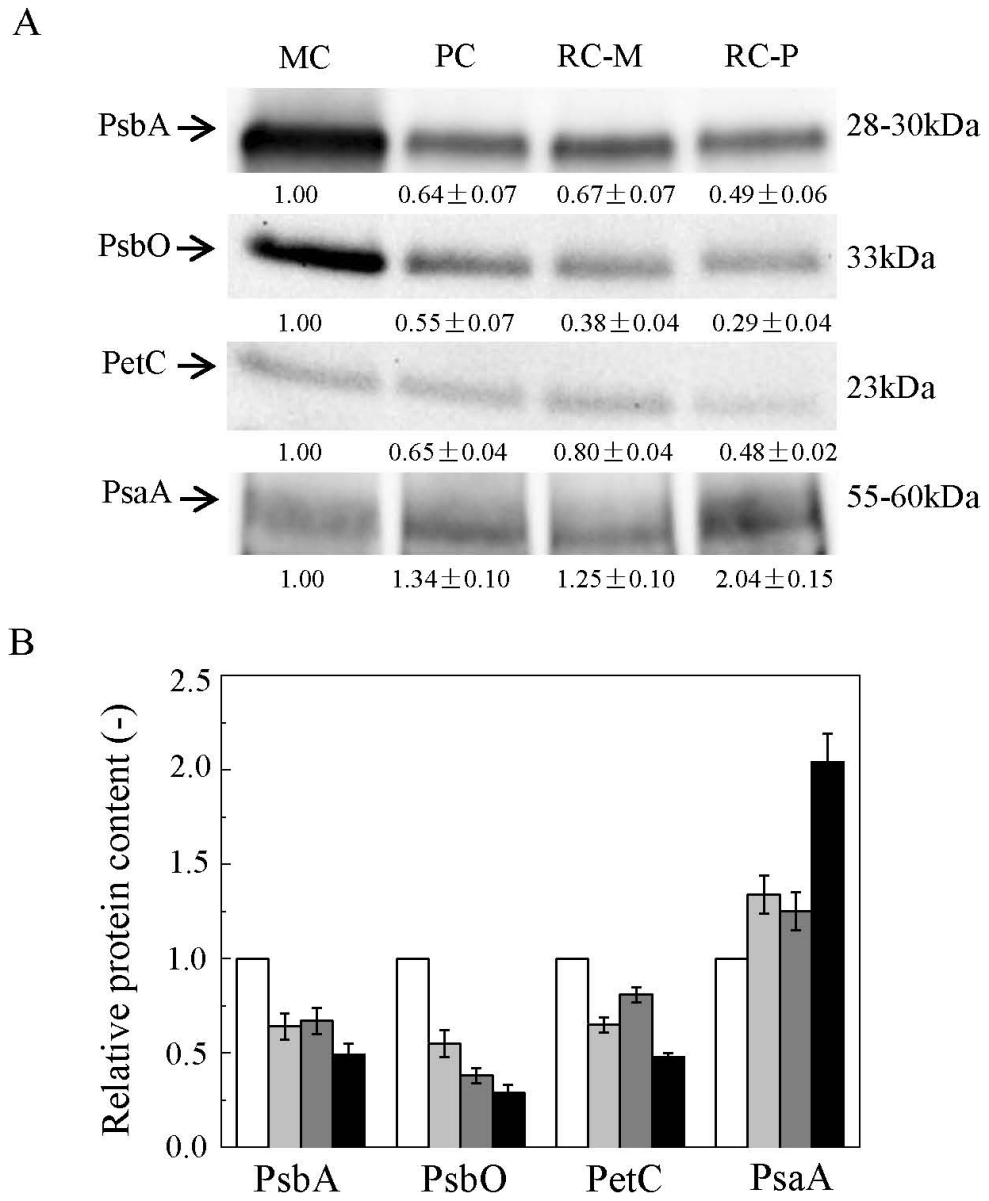
Analyses of photosystem protein content in different *H. pluvialis* cells types. (A) Western blot analyses on protein contents in different cells types; (B) relative protein content in different cells types from these analysis. Values represent the mean ± S.D. (n  =  2). MC: motile cells; PC: palmella cells; RC-M: red cells induced from motile cells; RC-P: red cells induced from palmella cells. MC: white rectangle; PC: light grey rectangle; RC-M: grey rectangle; RC-P: black rectangle.

Several components of the photosynthetic apparatuses in motile and palmella cells were quantified by western blotting (**Fig. 3A**). The content of D1 protein, the core reaction center protein of PSII, decreased 36% (*p*<0.05) during encystment under LL (**Fig. 3B**). Simultaneously, PsbO and PetC were reduced 45% (*p*<0.05) and 35% (*p*<0.05), respectively. In contrast to the attenuation of PSII and cytochrome *b*_6_ *f*, PsaA, a core reaction center protein of PSI, was 34% higher in palmella cells than in motile cells (*p*<0.05).

After exposure to HL for 24 h, D1 protein, PsbO, and PetC decreased in both motile and palmella cells to different extents (**Fig. 3A**). D1 and PsbO proteins in motile cells decreased 33% (*p*<0.05) and 62% (*p*<0.01), respectively, greater decreases than those seen in palmella cells during HL acclimation (23% and 47%, *p*<0.05, respectively) (**Fig. 3B**). As these PSII components are considered the primary targets of photosynthetically-produced ROS [28], these results indicate that motile cells are less capable of coping with photo-oxidative stress than palmella cells. Furthermore, after exposure to HL for 24 h, PsaA in palmella cells increased 52.2% (*p*<0.05), whereas remained unchanged in motile cells.

### Changes in biochemical composition during encystment and under high light

**Figure 4:**
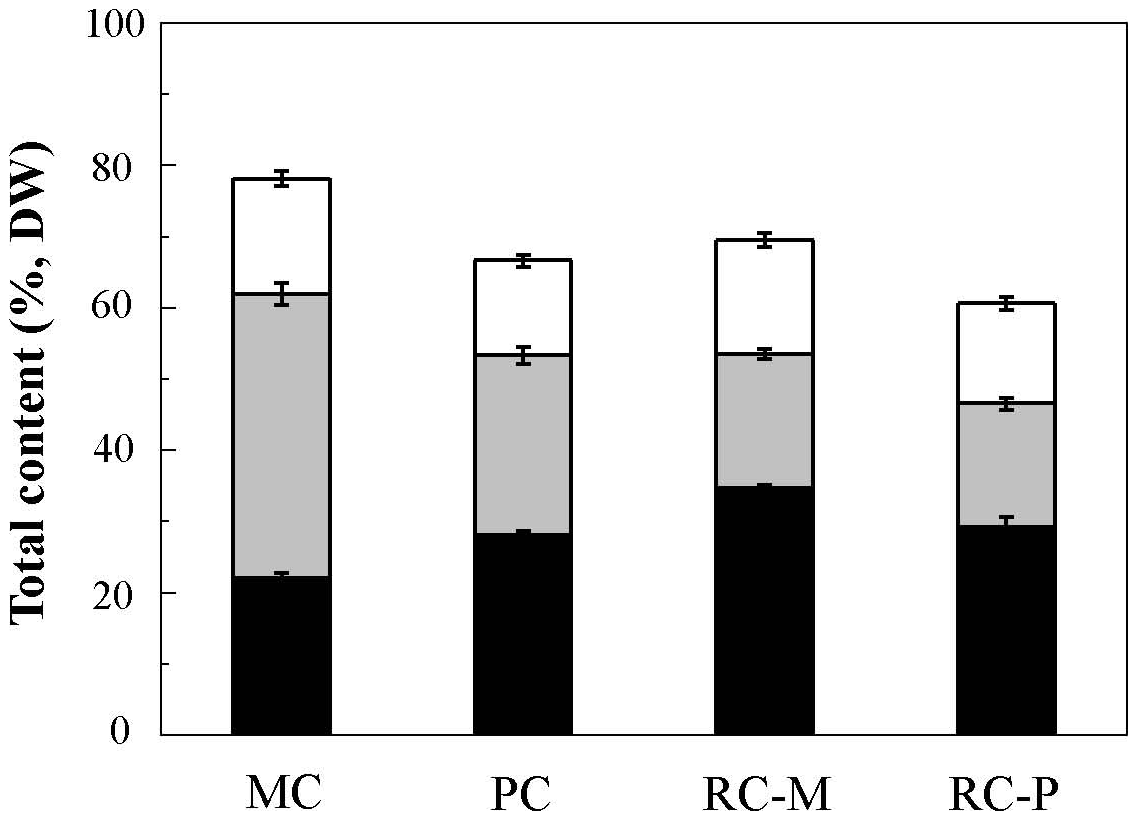
Analyses of total carbohydrate, protein, and glycerolipids in different *H. pluvialis* cell types. Values represent the mean ± S.D. (n = 6). MC: motile cells; PC: palmella cells; RC-M: red cells induced from motile cells; RC-P: red cells induced from palmella cells. Total glycerolipids: white rectangle; total protein: light grey rectangle; total carbohydrate: black rectangle.

The electrons produced by photosynthesis may be partitioned differentially into various biosynthetic pathways for synthesis of proteins, lipids, carbohydrates, and other molecules. The macromolecular composition of a given cell can then reflect the energy balance between absorbed photons and newly synthesized macromolecules or cell biomass [29], [30]. The biochemical compositions of the different cell forms were determined to compare their energy utilization efficiencies. As shown in **Fig. 4**, motile cells were composed of approximately 0.398 g g^−1^ DW protein, 0.221 g g^−1^ DW carbohydrate, and 0.162 g g^−1^ DW glycerolipid. The high protein content of the motile cells is in line with their high photosynthetic growth potential. The protein content in palmella cells was lower than in motile cells, and it decreased 36.7% (*p*<0.01) during the encystment process under LL. During encystment, carbohydrate content increased 27.5% (*p*<0.01), whereas glycerolipid content decreased 17.9% (*p*<0.05, **Fig. 4**). The increase in carbohydrates in palmella cells may be attributable to the accumulation of storage compounds, such as the cellulose associated with secondary cell walls [31].

After exposure to HL for 24 h, total protein content decreased 52.8% (*p*<0.01) and 31.2% (*p*<0.01) in motile and palmella cells, respectively (**Fig. 4**). Although the increase of lipids under HL was expected, as massive, TAG-rich lipid bodies were formed (**Fig. 1**), the total glycerolipid content in both palmella and motile cells remained essentially unchanged. At the same time, total carbohydrates in RC-M increased 57.0% (*p*<0.01) relative to the motile cells, whereas little change in carbohydrate content was observed in RC-P as compared to palmella cells. These results indicated that photosynthates were preferentially partitioned into storage carbohydrates (probably in the form of starch) in motile cells under HL.

### Quantitative analysis of membrane glycerolipids

**Figure 5:**
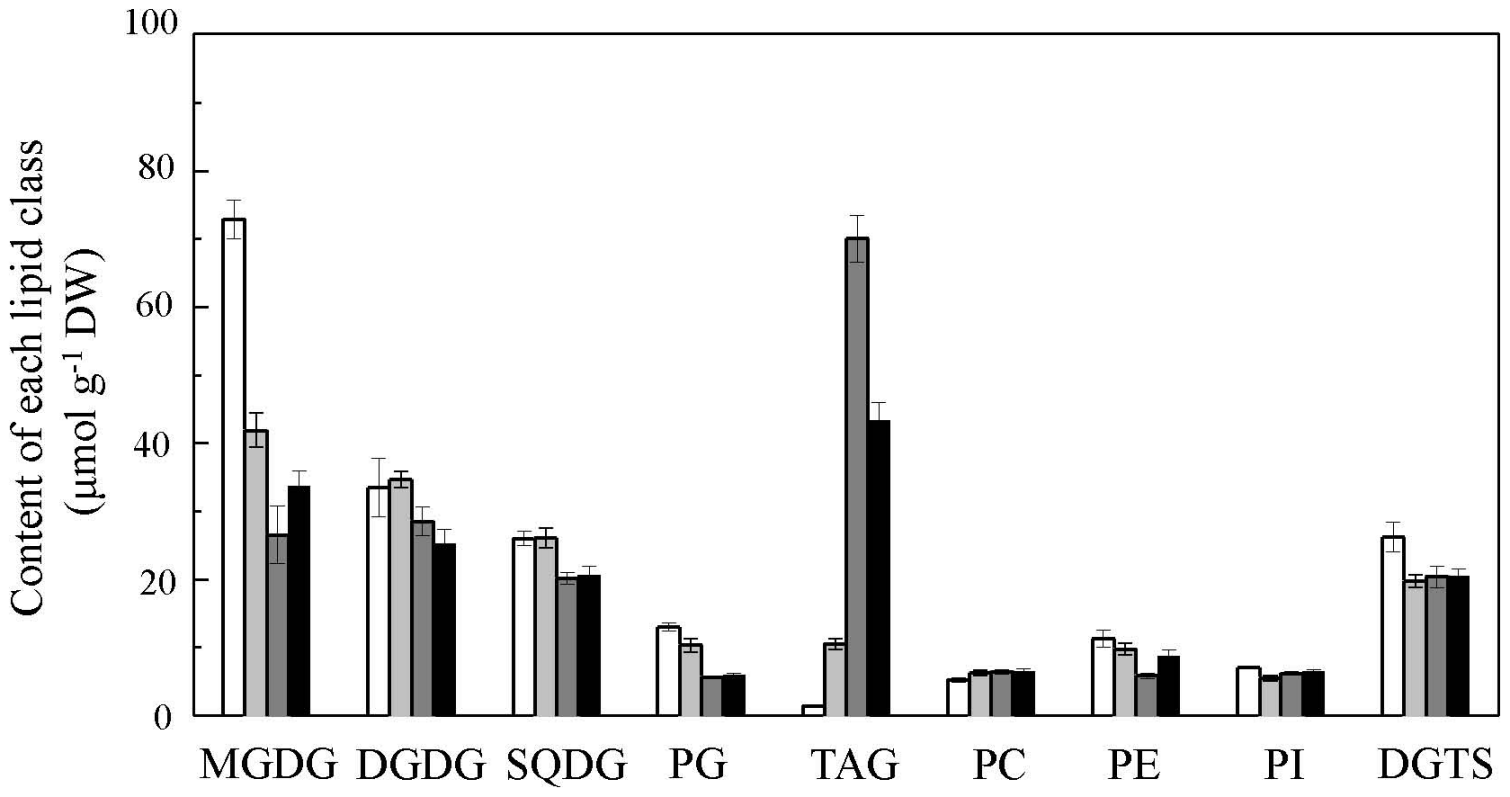
Content of different glycerolipid classes in different *H. pluvialis* cells types. Values represent the mean ± S.D. (n = 6). MC: motile cells; PC: palmella cells; RC-M: red cells induced from motile cells; RC-P: red cells induced from palmella cells. MC: white rectangle; PC: light grey rectangle; RC-M: grey rectangle; RC-P: black rectangle.

A lipidomics method was employed to quantitatively measure molecular species of glycerolipids in the different forms of *Haematococcus* cells. A total of eight classes of membrane glycerolipid (PC, PE, PI, PG, DGTS, MGDG, DGDG, and SQDG) were identified in all four types of *H. pluvialis* cells, i.e., motile cells, palmella cells, RC-M, and RC-P. The galactolipid MGDG was the most abundant membrane glycerolipid in motile and palmella cells grown under the favorable LL conditions, and the glycerolipid content in motile cells comprised up to 72.9 µmol g^−1^ DW, which was 42.5% greater than in palmella cells (41.9 µmol g^−1^ DW, *p*<0.01) (**Fig. 5**). The second most abundant galactolipids DGDG was ca. 50% of MGDG in motile and palmella cells. Such a ratio is similar to that found in many other microalgae and higher plants [32].

Four classes of phospholipids (PG, PC, PE, and PI) were detected in *Haematococcus* cells. PG, the only bulk phosphoglycerolipid found in thylakoid membranes [33], totaled about 6% of total glycerolipids in motile and palmella cells. Motile and palmella cells contained similar amounts of PE (ca. 11 µmol g^−1^ DW, accounting for 6% of total glycerolipids). During the encystment process under LL, PC increased 19.4% in palmella cells (6.27 µmol g^−1^ DW, *p*<0.01) compared to motile cells (5.25 µmol g^−1^ DW), whereas PI decreased from 7.05 µmol g^−1^ DW to 5.51 µmol g^−1^ DW during encystment.

Under HL, MGDG, DGDG, SQDG, and PG content in motile cells decreased 63.6% (*p*<0.01), 14.7% (*p*<0.05), 22.7% (*p*<0.01), and 56.4% (*p*<0.01), respectively, suggesting a dramatic degradation of the thylakoid membranes and photosynthetic complexes **(** **Fig. 5**). Palmella cells showed a similar reduction in DGDG, SQDG, and PG, but MGDG decreased only 19.4% under HL (*p*<0.01). In motile cells, PE and DGTS were reduced 47.9% (*p*<0.01) and 22.1% (*p*<0.05), respectively; however, both remained unchanged in palmella cells after 24 h under HL. In contrast to the remarkable reduction in chloroplast membrane lipids and nitrogen-containing glycerolipids (e.g., PE, DGTS), PI content increased 17.7% (*p*<0.01) in palmella cells during HL acclimation, and remained unchanged in motile cells. Among the eight membrane glycerolipid classes, PC was the only lipid that increased under HL, which occurred in motile cells (22.9%, *p*<0.01).

**Figure 6:**
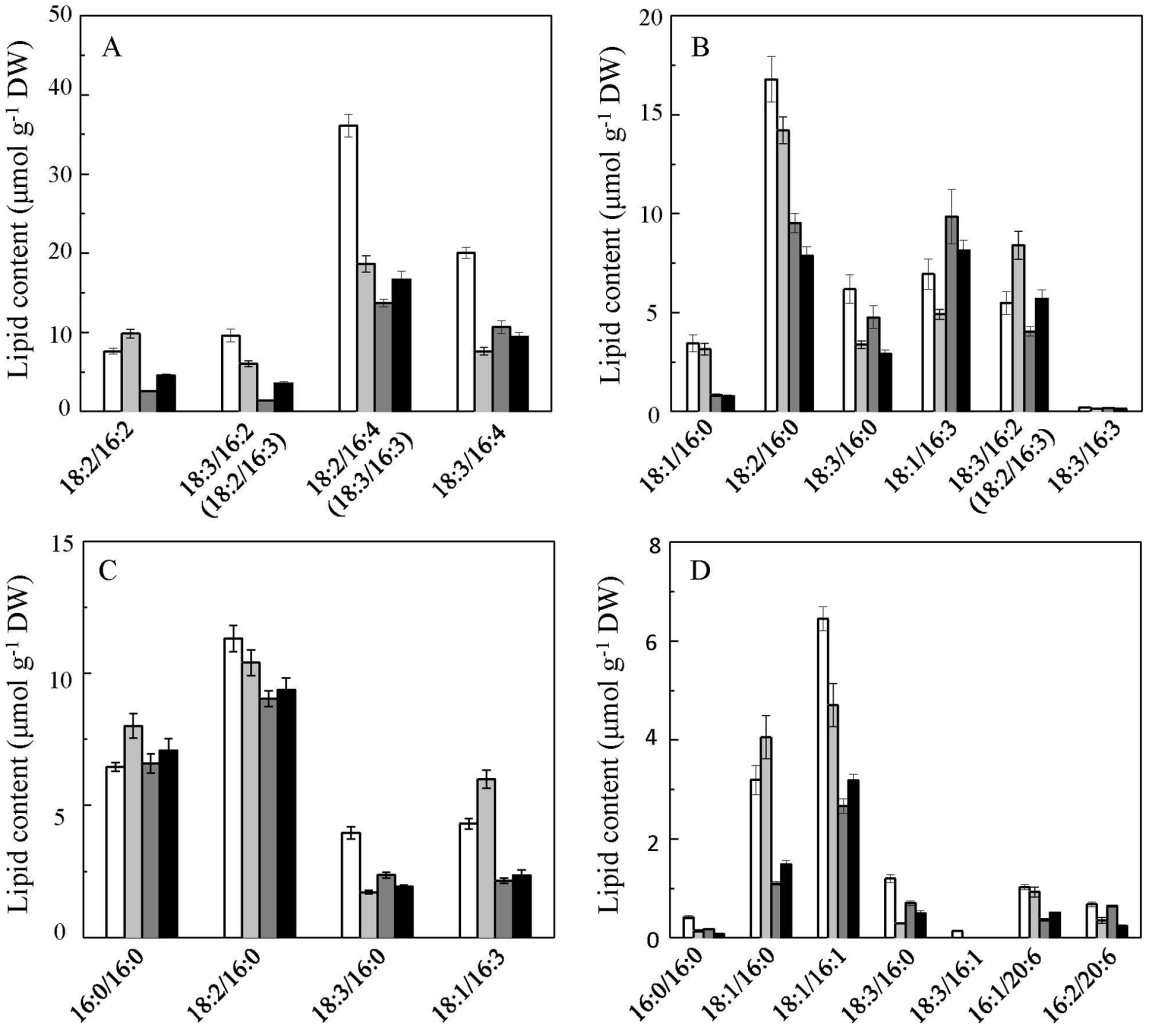
Lipid compositions of four major glycerolipids in *H. pluvialis* chloroplasts in different cells types. (A) MGDG; (B) DGDG; (C) SQDG; (D) PG. Values represent the mean ± S.D. (n = 6). MC: motile cells; PC: palmella cells; RC-M: red cells induced from motile cells; RC-P: red cells induced from palmella cells. MC: white rectangle; PC: light grey rectangle; RC-M: grey rectangle; RC-P: black rectangle.

**Figure 7:**
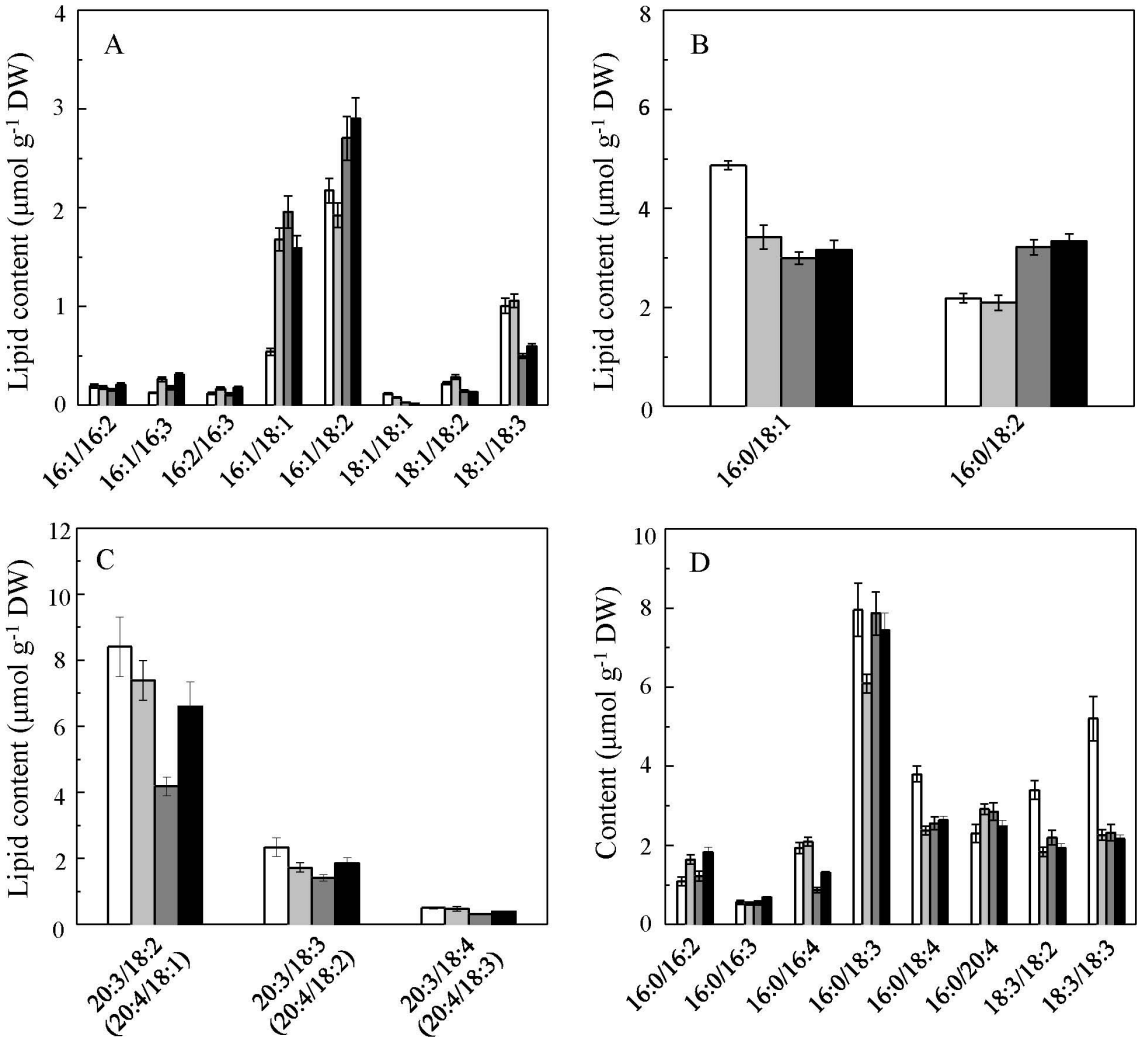
Lipid compositions of the extraplastidic glycerolipids in different *H. pluvialis* cells types. (A) PC; (B) PI; (C) PE; (D) DGTS. Values represent the mean ± S.D. (n = 6). MC: motile cells; PC: palmella cells; RC-M: red cells induced from motile cells; RC-P: red cells induced from palmella cells. MC: white rectangle; PC: light grey rectangle; RC-M: grey rectangle; RC-P: black rectangle.

The changes in cellular content of membrane glycerolipid molecules are shown in **Fig. 6** **and** **Fig. 7**. During the encystment process under LL, most chloroplast membrane glycerolipid molecules decreased. However, a few chloroplast membrane glycerolipids were up-regulated in palmella cells, including MGDG 34∶4, DGDG 34∶4, and SQDG 32∶0, among which DGDG 34∶4 showed the greatest change (a 53.3% increase in palmella cells, *p*<0.01). Under HL, a number of chloroplast membrane lipids, including MGDG (34∶5, 34∶6, and 34∶7), SQDG (34∶3), and PG (34∶3 and 34∶2), were reduced dramatically in motile cells but remained relatively stable in palmella cells (**Fig. 6**). A similar trend was observed with PI (34∶1), PE (38∶5, 38∶6), and DGTS (34∶4, 36∶5, and 36∶6) (**Fig. 7**).

### TAG profiling and quantitation

Although TAG synthesis is considered a protective strategy by which microalgae cope with environmental stress (e.g., nutrient deprivation, high light) [34], [35], our results reveal that *H. pluvialis*can accumulate TAG under favorable growing conditions. As shown in **Fig. 5** **,** motile cells contained a small but detectable amount of TAG (1.5 µmol g^−1^ DW), corresponding to 0.7% of total glycerolipids. During encystment under LL, TAG content increased ca. 7-fold in palmella cells (10.55 µmol g^−1^ DW, or 6.4% of total glycerolipids). Accumulation of TAG under favorable culture conditions has recently been reported in *Chlamydomonas* and *Nannochloropsis* as well [36].

When *H. pluvialis* cells were subjected to HL, the most noticeable change with respect to lipid composition was the accumulation of large amounts of TAG. TAG content in motile and palmella cells reached 70.04 and 43.41 µmol g^−1^ DW, respectively, after 24 h under HL, which accounted for 36.9% and 25.4% of total glycerolipids, respectively (**Fig. 5**).

**Figure 8:**
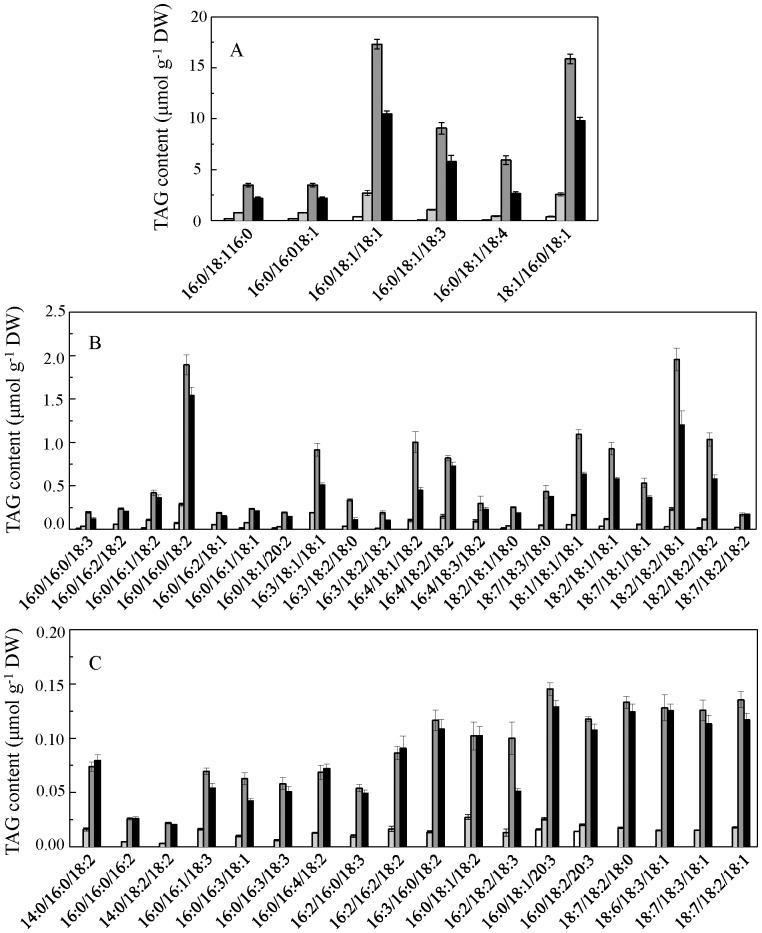
Triacylglycerol (TAG) composition in different *H. pluvialis* cells types. (A) Major species; (B) minor species; (C) trace species. Values represent the mean ± S.D. (n = 6). MC: motile cells; PC: palmella cells; RC-M: red cells induced from motile cells; RC-P: red cells induced from palmella cells. MC: white rectangle; PC: light grey rectangle; RC-M: grey rectangle; RC-P: black rectangle.

The cellular contents of individual TAG molecular species are shown in **Fig. 8**. The major TAG species under favorable growth conditions were TAG 50∶1, 52∶2, 52∶4, and 52∶5, which together accounted for more than 75% of total TAG in both motile and palmella cells. TAG (16∶0/18∶1/18∶1) and (18∶1/16∶0/18∶1) were the predominant species, accounting for ca. 25% of total TAG in both motile and palmella cells (**Fig. 8A**). Twenty-one TAG molecular species were present in minor quantities, accounting for 6.7–9.3% of total TAG (**Fig. 8B**), whereas eighteen TAG species combined to account for less than 3.5% of total TAG, and these eighteen TAGs were defined as trace TAG molecular species (**Fig. 8C**).

## Discussion

### Encystment process involves development of multiple defense mechanisms

When *H. pluvialis* cells were cultivated under favorable growth conditions for an extended period of time (e.g., 3–5 days), the motile cells lost their flagellae and became palmella cells with thickened cell walls. Our previous study [11] showed that motile and palmella cells are both capable of coping with environmental stress to different extents; however, the development of the protective mechanisms during encystment was not well understood. This study combined several physiological and biochemical tools to investigate several key photosynthetic and subcellular biochemical changes during the encystment.

The chlorophyll fluorometric analysis demonstrated that the capacity to dissipate excessive excited energy via the NPQ mechanism developed during encystment and was further augmented when palmella cells were subjected to HL. NPQ is a rapid and effective process that is induced seconds after photosynthetic cells are exposed to excess light [37]. When the excited energy exceeds the capacity of algal cells to use the reducing energy produced by photosynthesis for carbon fixation, algal cells can lower the quantum yield at PSII by dissipating the excess absorbed energy by NPQ. Thus, the development of NPQ in palmella cells may reduce the production of excessive ROS under HL conditions.

The adjustment of relative numbers of PSII and PSI complexes represents an important mechanism by which plants and algae prevent photodamage during high-light acclimation [38]–[40]. PSI cyclic electron transport may have multiple functions in photoprotection, such as dissipating energy absorbed at PSI and maintaining a ΔpH for NPQ to down-regulate energy production at PSII [26]. *H. pluvialis* cells changed the energy balance between PSII and PSI under HL by enhancing the quantum yield of PSI [Y(I)] while reducing Y(II) [15].We speculate that increasing the PSI/PSII ratio, decoupling PSI and PSII by decreasing cytochrome *b*_6_ *f* during encystment, and increasing cyclic electron transport around PSI are a suite of photoprotective mechanisms developed in palmella cells for acclimation under HL.

This study revealed for the first time the global remodeling of *H. pluvialis* glycerolipids in response to HL and under encystment. The ability of living cells to survive under extreme environmental conditions may rely on their ability to modify their membrane composition and adjust their lipid desaturation level [34], [41]. Prominent TAG accumulation, coupled with a reduction in the number of chloroplast lipid molecules species, was observed in palmella cells. TAG biosynthesis requires considerable amounts of reducing equivalents (NADPH), which may help relax over-reduced photosynthetic electron transport chains and thus protect the cells under stress [42]. In addition, TAG constitutes the storage subcellular structure (e.g., lipid bodies) for synthesized astaxanthin molecules in *H. pluvialis*, which can in turn provide protection from excess light [5], [14], [17], [43]. TAG may also be a depot of polyunsaturated fatty acids in some microalgae, allowing the organisms to swiftly adapt to the changing environment; the polyunsaturated fatty acids can be reincorporated into membrane lipids when environmental conditions become favorable for growth [34], [44].

Although production of ROS and cell mortality were not directly measured in this study, multiple lines of evidence suggest that the motile cells suffered more severe photo-oxidative stress than palmella cells when exposed to HL. First, more profound decreases in the quantum yields of PSII, D1 protein, and PsbO, as well as in several chloroplast membrane lipids (e.g., MGDG 18∶3/16∶2, 18∶2/16∶4, 18∶3/16:4) occurred in motile cells than in palmella cells under HL. In oxygenic photosynthetic organisms, PSII and PSI are two major sites of ROS production [26]. ROS produced at PSII and PSI can damage proteins, lipids, and pigments, especially D1 protein at PSII and lipids containing polyunsaturated fatty acids. Second, pronounced astaxanthin accumulation in motile cells is an indication of severe photo-oxidative stress induced under HL. Although astaxanthin can react with ROS and astaxanthin synthesis consumes molecular oxygen—the precursor of ROS—a higher astaxanthin content in motile cells than in palmella cells reflects a greater stress on motile cells exposed to HL. Consequently, motile cells may die off more quickly than cells with lesser amounts of astaxanthin; this was confirmed by our previous study in which *H. pluvialis* cells exposed to higher irradiance accumulated more astaxanthin but exhibited higher cell mortality [17].

### Remodeling of membrane glycerolipids under high light

Diacylglycerol-based polar lipids are the building blocks of the cellular membranes of living organisms. It is generally believed that glycolipids (e.g., MGDG, DGDG, SQDG) and the phospholipid PG are the major components of chloroplast thylakoid membranes, whereas phospholipids like PE, PC, PI, and the nonphosphorus betaine lipid DGTS reside in the extraplastidic membranes of photosynthetic cells [45]–[47].

Our results indicate that the major classes of chloroplast membrane lipids exhibit different fates under HL stress in *H. pluvialis*. PG showed the most profound decrease among all the chloroplast membrane lipids in both motile and palmella cells; MGDG was dramatically reduced in motile cells and red cysts under HL; by contrast, DGDG and SQDG showed moderate decreases in both motile and palmella cells under the same conditions. The different responses of these lipids may result from their uneven distributions among the photosynthetic complexes and their distinct functional roles in maintaining the proper structure and function of chloroplast membranes.

MGDG and DGDG are two major galactolipids that constitute the bilayer of thylakoid membranes. Moreover, MGDG has been identified in cyanobacterial PSII, PSI, and cytochrome *b*_6_ *f*, and DGDG is found in PSII, PSI and LHCII [48]–[50]. A sharp decrease of MGDG content in *H. pluvialis* cells is likely linked to the breakdown of PSII and cytochrome *b*_6_ *f* in response to HL, especially in motile cells. In the bilayer chloroplast membranes of *Arabidopsis*, MGDG can be converted to DGDG to prevent the formation of hexagonal structures and consequent membrane infusion under freezing stress [51]. Thus, the relatively stable amounts of DGDG in *H. pluvialis*under HL may be attributable in part to the conversion of MGDG to DGDG.

PGs are anionic lipids that are present primarily in PSII, PSI, and LHCII in *Thermosynechococcus elongates* and spinach (*Spinacia oleracea*) [33], [52]–[54]. In particular, PG may participate in the dimerization of PSII complexes and trimerization of PSI and LHCII complexes [33], [55]–[57]; it may also play a direct structural role in binding antenna pigments[58]. In this study, the observed drastic reduction of PG is likely involved in the breakdown of PSII, chlorophyll, and LHCII. SQDGs are another type of anionic lipid and are primarily associated with PSII and cytochrome *b*_6_ *f* complexes [58], [59]. SQDG may also partially replace PG to maintain the anionic surface charge of *Arabidopsis* thylakoid membranes under P_i_ starvation [60]–[62]. The moderate reduction of SQDG in both motile and palmella cells under nitrogen-depleted condition suggests that SQDG may be more stable than PG, thereby partially replacing PG while maintaining the anionic surface charge of the thylakoid membrane in *H. pluvialis* under HL.

### Biotechnical implications

Cell death during the first 1–2 days under photo-oxidative stress represents a major loss of biomass in *H. pluvialis* mass culture. This phenomenon often occurs in *Haematococcus* cells at the exponential growth phase, when the cell population is mainly in the flagellate form. Palmella cells can develop a suite of protective mechanisms during encystment to bestow greater resistance to HL than motile cells, and thus, applying palmella cells instead of motile cells to the stressful red stage of cultivation may represent a promising strategy for increasing growth and astaxanthin production. During the first day under HL, astaxanthin productivity (g L^−1^ day^−1^) in palmella cells was less than in motile cells. From a biotechnical perspective, high astaxanthin contents are desirable, although higher yields can be achieved be extending culturing time. Astaxanthin accumulation in palmella cells below the maximum potential may be attributable to the fact that palmella cells favor PSI cyclic electron transport over linear electron transport. Unlike linear electron transport, which produces both ATP and NADPH, cyclic electron transport is involved only in ATP production [63]. In *H. pluvialis* cells, 90% of astaxanthin is attached with one or two fatty acids [64], [65], forming astaxanthin mono- and diesters. Since NADPH is required for synthesis of astaxanthin and fatty acids [66], the reducing power of palmella cells may not be sufficient to produce amounts of astaxanthin esters equivalent to those produced by motile cells under the same circumstances.

Therefore, our future efforts will explore how to enhance NADPH production in palmella cells as well as how to increase the levels of astaxanthin biosynthetic enzymes through physical or genetic manipulations. Additionally, we will investigate biotic and abiotic factors that stimulate the development of protective mechanisms in palmella cells.

## References

[1] Lorenz RT, Cysewski GR (2000) Commercial potential for *Haematococcus* microalgae as a natural source of astaxanthin. Trends in Biotechnology 18: 160–167. 10740262 10.1016/s0167-7799(00)01433-5

[2] Guerin M, Huntley ME, Olaizola M (2003) *Haematococcus* astaxanthin: applications for human health and nutrition. Trends in Biotechnology 21: 210–216. 12727382 10.1016/S0167-7799(03)00078-7

[3] Boussiba S (2000) Carotenogenesis in the green alga *Haematococcus pluvialis*: cellular physiology and stress response. Physiologia Plantarum 108: 111–117.

[4] Han DX, Li YT, Hu Q (2013) Astaxanthin in microalgae: pathways, functions and biotechnological implications. Algae 28: 131–147.

[5] Lemoine Y, Schoefs B (2010) Secondary ketocarotenoid astaxanthin biosynthesis in algae: a multifunctional response to stress. Photosynthesis Research 106: 155–177. 20706789 10.1007/s11120-010-9583-3

[6] Damiani MC, Popovich CA, Constenla D, Leonardi PI (2010) Lipid analysis in *Haematococcus pluvialis* to assess its potential use as a biodiesel feedstock. Bioresource Technology 101: 3801–3807. 20117928 10.1016/j.biortech.2009.12.136

[7] Huntley M, Redalje D (2007) CO_2_ mitigation and renewable oil from photosysthetic microbes: a new appraisal. Mitigation and Adaptation Strategies for Global Change 12: 573–608.

[8] Aflalo C, Meshulam Y, Zarka A, Boussiba S (2007) On the relative efficiency of two- vs. one-stage production of astaxanthin by the green alga *Haematococcus pluvialis* . Biotechnology and Bioengineering 98: 300–305. 17318905 10.1002/bit.21391

[9] Harker M, Tsavalos AJ, Young AJ (1996) Autotrophic growth and carotenoid production of *Haematococcus pluvialis* in a 30 liter air-lift photobioreactor. Journal of Fermentation and Bioengineering 82: 113–118.

[10] Wang JF, Han DX, Sommerfeld MR, Lu CM, Hu Q (2013) Effect of initial biomass density on growth and astaxanthin production of *Haematococcus pluvialis* in an outdoor photobioreactor. Journal of Applied Phycology 25: 253–260.

[11] Han DX, Wang JF, Sommerfeld M, Hu Q (2012) Susceptibility and protective mechanisms of motile and non motile cells of *Haematococcus pluvialis* (Chlorophyceae) to photooxidative stress. Journal of Phycology 48: 693–705. 27011086 10.1111/j.1529-8817.2012.01147.x

[12] Hata N, Ogbonna JC, Hasegawa Y, Taroda H, Tanaka H (2001) Production of astaxanthin by *Haematococcus pluvialis* in a sequential heterotrophic-photoautotrophic culture. Journal of Applied Phycology 13: 395–402.

[13] Kobayashi M, Kakizono T, Nagai S (1993) Enhanced carotenoid biosynthesis by oxidative stress in acetate-induced cyst cells of a green unicellular alga, *Haematococcus pluvialis* . Applied and Environmental Microbiology 59: 867–873. 16348895 10.1128/aem.59.3.867-873.1993 PMC202201

[14] Li Y, Sommerfeld M, Chen F, Hu Q (2008) Consumption of oxygen by astaxanthin biosynthesis: a protective mechanism against oxidative stress in *Haematococcus pluvialis* (Chlorophyceae). Journal of Plant Physiology 165: 1783–1797. 18313796 10.1016/j.jplph.2007.12.007

[15] Gu WH, Xie XJ, Gao S, Zhou W, Pan GH, et al (2013) Comparison of different cells of *Haematococcus pluvialis* reveals an extensive acclimation mechanism during its aging process: from a perspective of photosynthesis. PLoS One 8: 1–10. 10.1371/journal.pone.0067028 PMC3724872 23922648

[16] Kobayashi M, Kakizono T, Nagai S (1991) Astaxanthin production by a green alga, *Haematococcus pluvialis* accompanied with morphological changes in acetate media. Journal of Fermentation and Bioengineering 71: 335–339.

[17] Li Y, Sommerfeld M, Chen F, Hu Q (2010) Effect of photon flux densities on regulation of carotenogenesis and cell viability of *Haematococcus pluvialis* (Chlorophyceae). Journal of Applied Phycology 22: 253–263. 20949119 10.1007/s10811-009-9453-6 PMC2946551

[18] Genty B, Briantais JM, Baker NR (1989) The relationship between the quantum yield of photosynthetic electron-transport and quenching of chlorophyll fluorescence. Biochimica et Biophysica Acta 990: 87–92.

[19] Kramer DM, Johnson G, Kiirats O, Edwards GE (2004) New fluorescence parameters for the determination of Q(A) redox state and excitation energy fluxes. Photosynthesis Research 79: 209–218. 16228395 10.1023/B:PRES.0000015391.99477.0d

[20] Eilers PHC, Peeters JCH (1988) A model for the relationship between light intensity and the rate of photosynthesis in phytoplankton. Ecological Modelling 42: 199–215.

[21] Rao P, Pattabiraman TN (1989) Reevaluation of the phenol sulfuric acid reaction for the estimation of hexoses and pentoses. Analytical Biochemistry 181: 18–22. 2817377 10.1016/0003-2697(89)90387-4

[22] Yoon K, Han DX, Li YT, Sommerfeld M, Hu Q (2012) Phospholipid: diacylglycerol acyltransferase Is a multifunctional enzyme involved in membrane lipid turnover and degradation while synthesizing triacylglycerol in the unicellular green microalga *Chlamydomonas reinhardtii* . Plant Cell 24: 3708–3724. 23012436 10.1105/tpc.112.100701 PMC3480297

[23] Welti R, Wang XM, Williams TD (2003) Electrospray ionization tandem mass spectrometry scan modes for plant chloroplast lipids. Analytical Biochemistry 314: 149–152. 12633615 10.1016/s0003-2697(02)00623-1

[24] Hsu FF, Turk J (2009) Electrospray ionization with low-energy collisionally activated dissociation tandem mass spectrometry of glycerophospholipids: mechanisms of fragmentation and structural characterization. Journal of Chromatography B-Analytical Technologies in the Biomedical and Life Sciences 877: 2673–2695. 10.1016/j.jchromb.2009.02.033 PMC2723218 19269264

[25] Han XL, Gross RW (2001) Quantitative analysis and molecular species fingerprinting of triacylglyceride molecular species directly from lipid extracts of biological samples by electrospray ionization tandem mass spectrometry. Analytical Biochemistry 295: 88–100. 11476549 10.1006/abio.2001.5178

[26] Niyogi KK (1999) Photoprotection revisited: genetic and molecular approaches. Annual Review of Plant Physiology and Plant Molecular Biology 50: 333–359. 10.1146/annurev.arplant.50.1.333 15012213

[27] Huang W, Yang SJ, Zhang SB, Zhang JL, Cao KF (2012) Cyclic electron flow plays an important role in photoprotection for the resurrection plant *Paraboea rufescens* under drought stress. Planta 235: 819–828. 22080919 10.1007/s00425-011-1544-3

[28] Aro EM, Virgin I, Andersson B (1993) Photoinhibition of photosystem II. Inactivation, protein damage and turnover. Biochimica et Biophysica Acta 1143: 113–134. 8318516 10.1016/0005-2728(93)90134-2

[29] Jakob T, Wagner H, Stehfest K, Wilhelm C (2007) A complete energy balance from photons to new biomass reveals a light- and nutrient-dependent variability in the metabolic costs of carbon assimilation. Journal of Experimental Botany 58: 2101–2112. 17483116 10.1093/jxb/erm084

[30] Langner U, Jakob T, Stehfest K, Wilhelm C (2009) An energy balance from absorbed photons to new biomass for *Chlamydomonas reinhardtii* and *Chlamydomonas acidophila* under neutral and extremely acidic growth conditions. Plant Cell and Environment 32: 250–258. 10.1111/j.1365-3040.2008.01917.x 19054351

[31] Taylor NG, Scheible WR, Cutler S, Somerville CR, Turner SR (1999) The irregular xylem3 locus of *Arabidopsis* encodes a cellulose synthase required for secondary cell wall synthesis. Plant Cell 11: 769–779. 10330464 10.1105/tpc.11.5.769 PMC144224

[32] Shimojima M, Ohta H (2011) Critical regulation of galactolipid synthesis controls membrane differentiation and remodeling in distinct plant organs and following environmental changes. Progress in Lipid Research 50: 258–266. 21414359 10.1016/j.plipres.2011.03.001

[33] Wada H, Murata N (2007) The essential role of phosphatidylglycerol in photosynthesis. Photosynthesis Research 92: 205–215. 17634751 10.1007/s11120-007-9203-z

[34] Merzlyak MN, Chivkunova OB, Gorelova OA, Reshetnikova IV, Solovchenko AE, et al (2007) Effect of nitrogen starvation on optical properties, pigments, and arachidonic acid content of the unicellular green alga *Parietochloris incisa* (Trebouxiophyceae, Chlorophyta). Journal of Phycology 43: 833–843.

[35] Solovchenko AE (2012) Physiological role of neutral lipid accumulation in eukaryotic microalgae under Stresses. Russian Journal of Plant Physiology 59: 167–176.

[36] Liu BS, Vieler A, Li C, Jones AD, Benning C (2013) Triacylglycerol profiling of microalgae *Chlamydomonas reinhardtii* and *Nannochloropsis oceanica* . Bioresource Technology 146: 310–316. 23948268 10.1016/j.biortech.2013.07.088

[37] Kulheim C, Agren J, Jansson S (2002) Rapid regulation of light harvesting and plant fitness in the field. Science 297: 91–93. 12098696 10.1126/science.1072359

[38] Allorent G, Tokutsu R, Roach T, Peers G, Cardol P, et al (2013) A dual strategy to cope with high light in *Chlamydomonas reinhardtii* . Plant Cell 25: 545–557. 23424243 10.1105/tpc.112.108274 PMC3608777

[39] Allen JF (1992) Protein-phosphorylation in regulation of photosynthesis. Biochimica et Biophysica Acta 1098: 275–335. 1310622 10.1016/s0005-2728(09)91014-3

[40] Delosme R, Olive J, Wollman FA (1996) Changes in light energy distribution upon state transitions: an in vivo photoacoustic study of the wild type and photosynthesis mutants from *Chlamydomonas reinhardtii* . Biochimica et Biophysica Acta-Bioenergetics 1273: 150–158.

[41] Thompson GA (1996) Lipids and membrane function in green algae. Biochimica et Biophysica Acta-Lipids and Lipid Metabolism 1302: 17–45. 10.1016/0005-2760(96)00045-8 8695653

[42] Roessler PG (1990) Environmental control of glycerolipid metabolism in microalgae commercial implications and future research directions. Journal of Phycology 26: 393–399.

[43] Zhekisheva M, Zarka A, Khozin-Goldberg I, Cohen Z, Boussiba S (2005) Inhibition of astaxanthin synthesis under high irradiance does not abolish triacylglycerol accumulation in the green alga *Haematococcus pluvialis* (Chlorophyceae). Journal of Phycology 41: 819–826.

[44] Khozin-Goldberg I, Shrestha P, Cohen Z (2005) Mobilization of arachidonyl moieties from triacylglycerols into chloroplastic lipids following recovery from nitrogen starvation of the microalga *Parietochloris incisa* . Biochimica et Biophysica Acta-Molecular and Cell Biology of Lipids 1738: 63–71. 10.1016/j.bbalip.2005.09.005 16324884

[45] Kobayashi K, Kondo M, Fukuda H, Nishimura M, Ohta H (2007) Galactolipid synthesis in chloroplast inner envelope is essential for proper thylakoid biogenesis, photosynthesis, and embryogenesis. Proceedings of the National Academy of Sciences of the United States of America 104: 17216–17221. 17940034 10.1073/pnas.0704680104 PMC2040463

[46] Mizusawa N, Sakurai I, Kubota H, Wada H (2007) Role of phosphatidylglycerol in oxygen-evolving complex of photosystem II. Photosynthesis Research 91: 175–175.

[47] Ohlrogge J, Browse J (1995) Lipid biosynthesis. Plant Cell 7: 957–970. 7640528 10.1105/tpc.7.7.957 PMC160893

[48] Holzl G, Zahringer U, Warnecke D, Heinz E (2005) Glycoengineering of cyanobacterial thylakoid membranes for future studies on the role of glycolipids in photosynthesis. Plant and Cell Physiology 46: 1766–1778. 16120686 10.1093/pcp/pci189

[49] Steffen R, Kelly AA, Huyer J, Dormann P, Renger G (2005) Investigations on the reaction pattern of photosystem II in leaves from *Arabidopsis thaliana* wild type plants and mutants with genetically modified lipid content. Biochemistry 44: 3134–3142. 15736923 10.1021/bi048465f

[50] Reifarth F, Christen G, Seeliger AG, Dormann P, Benning C, et al (1997) Modification of the water oxidizing complex in leaves of the dgd1 mutant of *Arabidopsis thaliana* deficient in the galactolipid digalactosyldiacylglycerol. Biochemistry 36: 11769–11776. 9305967 10.1021/bi9709654

[51] Moellering ER, Muthan B, Benning C (2010) Freezing tolerance in plants requires lipid remodeling at the outer chloroplast membrane. Science 330: 226–228. 20798281 10.1126/science.1191803

[52] Jordan P, Fromme P, Witt HT, Klukas O, Saenger W, et al (2001) Three-dimensional structure of cyanobacterial photosystem I at 2.5 angstrom resolution. Nature 411: 909–917. 11418848 10.1038/35082000

[53] Loll B, Kern J, Saenger W, Zouni A, Biesiadka J (2005) Towards complete cofactor arrangement in the 3.0 angstrom resolution structure of photosystem II. Nature 438: 1040–1044. 16355230 10.1038/nature04224

[54] Liu ZF, Yan HC, Wang KB, Kuang TY, Zhang JP, et al (2004) Crystal structure of spinach major light-harvesting complex at 2.72 angstrom resolution. Nature 428: 287–292. 15029188 10.1038/nature02373

[55] El Maanni A, Dubertret G, Delrieu MJ, Roche O, Tremolieres A (1998) Mutants of *Chlamydomonas reinhardtii* affected in phosphatidylglycerol metabolism and thylakoid biogenesis. Plant Physiology and Biochemistry 36: 609–619.

[56] Sakurai I, Hagio M, Gombos Z, Tyystjarvi T, Paakkarinen V, et al (2003) Requirement of phosphatidylglycerol for maintenance of photosynthetic machinery. Plant Physiology 133: 1376–1384. 14551333 10.1104/pp.103.026955 PMC281632

[57] Dubertret G, Gerard-Hirne C, Tremolieres A (2002) Importance of trans-Delta(3)-hexadecenoic acid containing phosphatidylglycerol in the formation of the trimeric light-harvesting complex in *Chlamydomonas* . Plant Physiology and Biochemistry 40: 829–836.

[58] Jones MR (2007) Lipids in photosynthetic reaction centres: structural roles and functional holes. Progress in Lipid Research 46: 56–87. 16963124 10.1016/j.plipres.2006.06.001

[59] Shimojima M (2011) Biosynthesis and functions of the plant sulfolipid. Progress in Lipid Research 50: 234–239. 21371504 10.1016/j.plipres.2011.02.003

[60] Benning C, Beatty JT, Prince RC, Somerville CR (1993) The sulfolipid sulfoquinovosyldiacylglycerol is not required for photosynthetic electron-transport in rhodobacter-sphaeroides but enhances growth under phosphate limitation. Proceedings of the National Academy of Sciences of the United States of America 90: 1561–1565. 8434018 10.1073/pnas.90.4.1561 PMC45914

[61] Sato N (2004) Roles of the acidic lipids sulfoquinovosyl diacylglycerol and phosphatidylglycerol in photosynthesis: their specificity and evolution. Journal of Plant Research 117: 495–505. 15538651 10.1007/s10265-004-0183-1

[62] Yu B, Benning C (2003) Anionic lipids are required for chloroplast structure and function in *Arabidopsis* . Plant Journal 36: 762–770. 14675442 10.1046/j.1365-313x.2003.01918.x

[63] Newo R, Chuartzman SG, Tsabari O, Reich Z, Charuvi D, et al.. (2009) Architecture of thylakoid membrane networks. In: Wada H, Murata N, editors. Lipids in photosynthesis: essential and regulatory functions. 1st ed. Dordrecht: Springer. pp. 295–328.

[64] Grung M, Dsouza FML, Borowitzka M, Liaaenjensen S (1992) Algal carotenoids 51. secondary carotenoids 2. *Haematococcus pluvialis* aplanospores as a source of (3s, 3′s)-astaxanthin esters. Journal of Applied Phycology 4: 165–171.

[65] Miao FP, Lu DY, Li YG, Zeng MT (2006) Characterization of astaxanthin esters in *Haematococcus pluvialis* by liquid chromatography-atmospheric pressure chemical ionization mass spectrometry. Analytical Biochemistry 352: 176–181. 16597431 10.1016/j.ab.2006.03.006

[66] Chumpolkulwong N, Kakizono T, Ishii H, Nishio N (1997) Enzymatic conversion of beta-carotene to astaxanthin by cell-extracts of a green alga *Haematococcus pluvialis* . Biotechnology Letters 19: 443–446.

